# Preclinical study of an optimized AAV cancer vaccine in a spontaneous canine model of oral melanoma

**DOI:** 10.1016/j.omton.2026.201282

**Published:** 2026-06-26

**Authors:** Federica Ghersa, Caitlin Yung, Ester Molina, Hisae Kuoch, Colin Caine, Karina Krotova, Antonella Borgatti, George Aslanidi

**Affiliations:** 1 The Hormel Institute, University of Minnesota, Austin, MN 55912, USA; 2 University of Minnesota College of Veterinary Medicine, St Paul, MN 55108, USA; 3 Clinical Investigation Center, St Paul, MN 55108, USA; 4 Masonic Cancer Center, Minneapolis, MN 55455, USA; 5 Center for Genomic Engineering, Minneapolis, MN 55455, USA

**Keywords:** adeno-associated virus, canine model, cancer vaccine, antigen-specific T cells, protective immune response

## Abstract

Like humans, companion dogs develop cancers spontaneously in the context of increased age, genetic variations, or environmental exposures, making them valuable as a preclinical model. Here, we assessed the safety and preliminary effectiveness of a multivalent adeno-associated virus (AAV) cancer vaccine in spontaneous canine oral melanoma. Eight companion dogs were vaccinated with equal doses of three different optimized AAV vectors encoding the melanoma others glycoprotein 100 (GP100), tyrosinase (Tyr), and tyrosinase-related protein 1 (TRP-1). Five dogs received a dose of 3 × 10^12^ viral genomes (vg)/dog, and three others received 9 × 10^12^ vg/dog. Post-vaccination monitoring established a lack of adverse effects and confirmed the presence of antigen-specific IFN-γ-producing T cells in the blood of most dogs. Although the variable survival times make it difficult to evaluate the effectiveness of the optimized AAV cancer vaccine at this point, three of the eight dogs were disease-free by the end of study, and two dogs survived 804 and 953 days after vaccination, and one is currently alive. Thus, the optimized AAV cancer vaccine showed promise in a spontaneous cancer animal model that closely recapitulates human disease development.

## Graphical abstract

**Figure:**
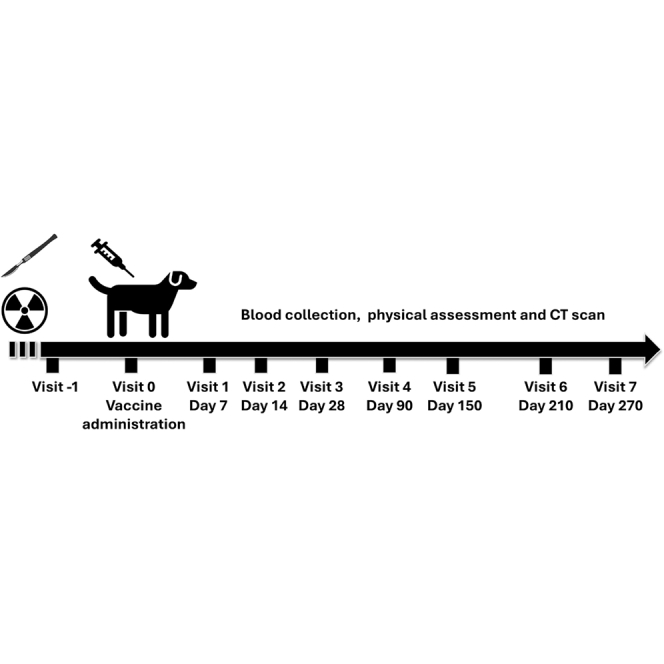

## Teaser

Aslanidi and colleagues evaluates the safety and efficacy of an AAV-based cancer vaccine to treat oral melanoma diagnosed in companion dogs. The results indicate that vaccine is well tolerated without side effects and 3 out of 8 dogs were disease free by the end of study, indicating a promising treatment option.

## Introduction

Adeno-associated viruses (AAVs) have been proven to be safe and efficient vectors in preclinical and clinical trials in the last few decades, with growing number of AAV-based treatments approved by the United States (US) Food and Drug Administration (FDA) or equivalent agencies in Europe, the United Kingdom, Canada, and China for monogenic diseases. At the same time, recent evidence obtained from numerous human clinical trials overturned the concept that AAV vectors have low immunogenicity^1^^,^^2^^,^^3^ and facilitated the expansion of AAV in new areas of use as immunomodulatory treatments for complex illnesses, particularly cancers and infectious diseases.

A major rationale for repurposing AAV as a vaccine platform is its strong safety record from gene therapy: AAVs are non-pathogenic, extensively studied clinically, and typically require high doses only when large numbers of cells must be transduced, whereas vaccines can rely on transduction of relatively small number of target cells, such as residential dendritic cells (DC), to elicit protective immunity.^4^^,^^5^^,^^6^^,^^7^^,^^8^^,^^9^ Practical features also favor AAV, which is stable at 4°C and at room temperature (RT), unlike mRNA, reducing cold-chain requirements and facilitating deployment in low-resource settings despite current manufacturing costs.^6^^,^^10^^,^^11^^,^^12^^,^^13^

Various AAV platforms are now being engineered as cancer vaccines to generate durable, tumor-specific immune responses, primarily in murine models targeting neoantigens, tumor-associated antigens, or viral/foreign antigens expressed by tumors.^14^^,^^15^^,^^16^^,^^17^^,^^18^^,^^19^^,^^20^^,^^21^ Preclinical studies on cancer mouse models show that AAV-based vaccines can induce durable immunity and permit flexible antigen design.^10^^,^^11^^,^^12^^,^^15^^,^^22^^,^^23^ Two main formats have emerged: gene-based vaccines, in which AAV delivers an antigen-encoding cassette under a strong promoter to drive heightened *in vivo* expression and durable antibody and T cells responses,^10^^,^^11^^,^^13^^,^^22^ and capsid-display or virus-like particle approaches, where epitopes or protein domains are mutated or inserted into capsid proteins to create highly repetitive antigen arrays that potently stimulate B cells activation and memory.^12^^,^^15^^,^^22^^,^^23^

In a gene-based AAV vaccine composed of AAV6 with the capsid modification S663V that ensures double-stranded DNA cargo delivery to the nucleus by avoiding proteasomal degradation. This AAV vector carried the truncated tumor antigens glycoprotein 100 (GP100), tyrosinase (Tyr), or tyrosinase-related protein 1 (TRP-1) in an optimized expression cassette with SEC (MHC class I leader sequence)and MITD ( MHC class I trafficking signal sequence) that exploited the DC antigen processing machinery inducing CD8 ^+^ and CD4^+^ T cells response. We demonstrated the efficiency of our vaccine in mouse models of B16F10 melanoma and prolonging animal survival.^16^^,^^17^^,^^24^

Dogs develop spontaneous tumors with genetic similarities to human tumors, usually as a result of environmental conditions, genetic defects, or advanced age, as happens in humans,^25^ making them an ideal model for cancer and other complex diseases.^26^^,^^27^^,^^28^^,^^29^^,^^30^^,^^31^ Additional advantages include the shared home environment and risk factors with owners, which increase relevance for chronic multifactorial conditions, and shorter lifespans with more rapid disease courses, which enable faster therapeutic readouts.^27^^,^^30^^,^^31^^,^^32^ Canine patients also allow evaluation of dosing, toxicity, and immunotherapies in immune-competent, tumor-bearing subjects.^27^^,^^29^^,^^31^^,^^33^

Moreover, dogs have already provided direct translational paths to human trials and there are several AAV-based therapies currently approved by the FDA that were tested in dogs as a preclinical model; among these are treatments for Leber congenital amaurosis,^34^^,^^35^ hemophilia A and B,^36^^,^^37^^,^^38^^,^^39^^,^^40^ and muscular dystrophy.^41^^,^^42^ Currently, these studies have expanded to include cancer immune^43^^,^^44^ and oncolytic virus^45^^,^^46^ therapies. The genetic similarities between human and canine tumors and the high resemblance of the dog immune system to the human immune system, make dogs a suitable preclinical model for cancer immunotherapy. Particularly relevant to our work, is the example of a preclinical study of the treatment of canine malignancies with autologous DCs loaded *ex vivo* with GP100 to activate the immune system of the dogs and reduce the tumor growth.^47^ Another strategy for treating oral melanoma in dogs, is the use of a DNA vaccine (Oncept) carrying Tyr, which is currently approved to use in veterinary practice.^37^ Additionally, the monoclonal antibody, anti-PD-1 is being developed for improvement of T cells function in dogs.^48^

Canine oral melanoma, like humans, is a very aggressive disease that often metastasizes, which reduces the survival rate of the animals.^49^^,^^50^ We hypothesized that our optimized multivalent (Gp100, Tyr, and TRP-1) vaccine in combination with standard treatments such as surgery and radio therapy will be effective in controlling tumor recurrence and metastatic spread to the lungs. The objectives of our study were (1) assess the potential toxicity in response to AAV vaccine administration; (2) evaluate the generation of systemic immune responses to each antigen(s) following delivery via intramuscular injection of AAV vaccine; including the kinetics, duration, and magnitude of these immune responses; and (3) obtain preliminary evidence of vaccine’s ability to suppress the development of lung metastasis and improve the long-term overall survival of the dogs without adverse side effects.

Animals were followed for 9 months after single intramuscular injection of AAV vaccine and analyzed for blood biochemistry, hematological parameters, chest radiography, and the immune reaction against both the viral capsid and the encoded antigen(s). No adverse reaction was documented in any dogs, and 6 out of 8 animals presented clear evidence of IFN-γ production by peripheral blood mononuclear cells (PBMCs). The results from our study indicate that optimized AAV vaccine based on 3 off-shelf tumor antigens is safe in dogs and preliminary efficacy was achieved on several animals.

## Results

### Patient population and disease staging

Eight dogs out of twelve screened were enrolled in the clinical trial, five males and three females with ages between 6.5 and 14.3 years. Two groups were vaccinated intramuscularly with 3 × 10^12^ viral genome (vg)/dog or 9 × 10^12^ vg/dog. Of the eight dogs, three completed all eight scheduled visits after vaccination, and five dogs were excluded owing to disease progression or other complications. Five dogs were vaccinated within 3 months after diagnosis, and three dogs were vaccinated more than 3 months after diagnosis because of further evaluations or treatments such as surgical removal of the tumor and radiotherapy before vaccination.

Disease staging was evaluated by computed tomography (CT) for skull, neck, and thorax or thoracic radiographs for local recurrence and distant metastasis in lungs. Locoregional lymphadenopathy (including mandibular, retropharyngeal, and prescapular) was detected on CT in 4/6 dogs (66.7%) dogs and none of dogs revealed evidence of pulmonary involvement. Additionally, dogs had a fine needle aspiration of both mandibular and prescapular lymph nodes. None of the lymph nodes contralateral to each dog’s tumor had overt evidence of metastasis on cytologic review.

In contrast, three dogs (37.5%) had evidence of metastasis ipsilateral to the site of their melanoma. One dog (12.5%) had its ipsilateral lymph node return as reactive to radiation that was performed prior to screening versus true melanoma metastasis. One dog (12.5%) had its ipsilateral lymph node return as non-diagnostic. Two patients had other forms of staging performed, including an abdominal ultrasound performed by a radiologist (1/8) and mandibular and retropharyngeal lymph node extirpation (1/8). Data on diseases staging, age, weight, doses, local therapy, and overall survival are summarized in Table 1.

**Table 1:** Summary table of the data of the patients enrolled in the study

Patient data	ID	Age (years)	Breed	Sex & Neuter status	Weight (kg)	Date of diagnosis	World Health Organization (WHO) stage prior to local therapy	Therapy prior to vaccine	Date(s) of local therapy	Date of injection	Overall survival (days)
Low dose (3 × 10^12^ vg/dog)	A001	14.3	Beagle	MN	10.5	01.04.2022	II	radiation therapy (4 × 6Gy)	03.02.22–03.23.22	03.31.22	238
	A002	13.1	Labrador retriever	MN	32.2	01.26.2022	II	caudal mandibulectomy	01.26.22	06.07.22	238
	A003	10.2	Goldendoodle	MN	31.4	04.12.2022	II	definitive intent radiation therapy (5 × 5Gy)	05.11.22–05.17.22	06.07.22	953
	A004	12.2	mixed breed	FS	10.2	06.21.2022	I	complete narrow surgical excision	06.21.22	08.25.22	804
	AA06	11.0	Miniature poodle	MN	6.2	02.09.2023	III	incomplete surgical excision (twice)	10.17.22 and 04.12.23	05.08.23	579
High dose (9 × 10^12^ vg/dog)	AA05	10.1	Dachshund	FS	11.1	10.17.2022	III	incomplete surgical excision	02.09.23	05.08.23	132
	AA07	6.5	Bluetick hound	MN	34.0	05.09.2023	I	complete narrow surgical excision then incomplete surgical excision of recurrence	05.09.22 and 04.25.23	05.22.23	462
	AA08	7.9	Havanese	FS	6.3	04.17.2023	I	complete narrow surgical excision and revision surgery	04.17.23 and 09.06.23	09.19.23	895 (alive)

A total of five males and three females were enrolled in the study in two different cohorts: the low-dose cohort was injected with a total of 3 × 10^12^ viral genomes (vg), and the high-dose cohort was injected with 9 × 10^12^ vg. The age of the dogs’ ranged between 14.3 and 6.5 years, with a median of 11 years. The weights ranged between 34 and 6.2 kg with a median of 11.1 kg. The survival number of days is calculated from the injection date until date of manuscript submission. Stage of oral melanoma, and treatments prior vaccine administration, and dates of treatments are also shown for each patient.

### Induction of early inflammatory markers

**Figure 1:**
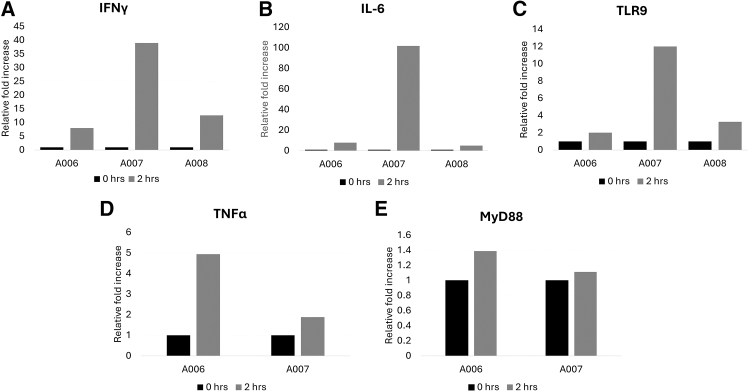
Expression of inflammatory marker in dog`s PBMCs before and after 2 h post vaccination RNA was extracted from PBMC collected before treatment and 2 h post-vaccination. Inflammatory markers IFN-γ (A), IL-6 (B), TLR9 (C), TNF-α (D), and MyD88 (E) were assessed by qPCR in three dogs. Data are presented as relative fold increase after treatment compared to pre-treatment levels which were assigned as 1. There was a trend in upregulation of the inflammatory markers after vaccination in all analyzed dogs. The statistical significance was not obtained because of one time collection of corresponding samples.

No dog displayed any adverse events in the 2 h following vaccination. PBMCs collected from dogs AA06, AA07, and AA08 at 2 h after vaccination were used to evaluate inflammatory markers previously identified as early responses to AAV injection (MyD88, TLR9, IFN-γ, TNF-α, and IL-6) (Figure 1). The results obtained by qPCR showed an increase of inflammatory markers 2 h after vaccination, indicating that vaccination did indeed induce a mild inflammatory immune response, similar to findings in human clinical trials that have shown a peak of inflammatory cytokines provoked by mRNA vaccine as soon as 2 h after vaccination.^17^

### Inflammatory response

**Figure 2:**
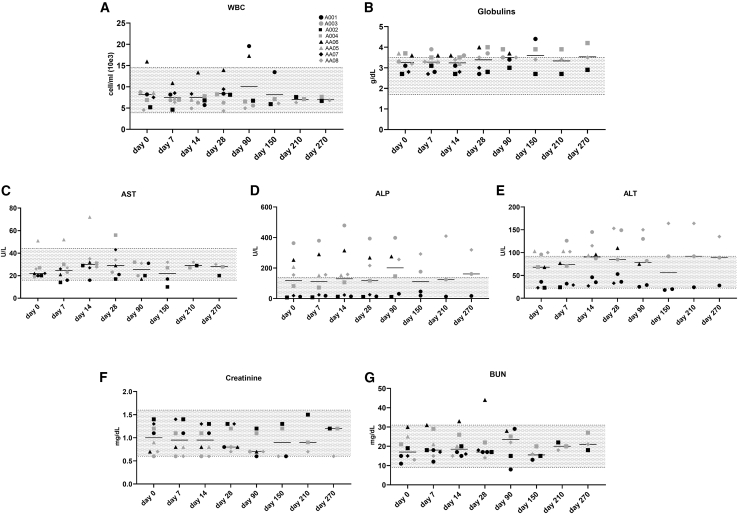
Kinetics of biochemical and hematological parameters analyzed throughout the study White blood cell (WBC) count (A) and globulin concentration (B) were used as indicators of the immune response. Potential liver toxicity was evaluated by measuring the activity of liver enzymes aspartate aminotransferase (AST) (C), alkaline phosphatase (ALP) (D), and alanine aminotransferase (ALT) (E). Creatinine (F) and blood urea nitrogen (BUN) (G) parameters were assessed to evaluate possible renal toxicity. Symbols on the graphs represent values from individual dogs. The shaded areas indicate the normal reference ranges. WBC counts are presented as thousands of cells per μL. Globulin concentrations are expressed in g/dL. Liver enzyme activities are reported in units per liter (U/L). Creatinine and BUN concentrations are expressed in mg/dL.

The inflammatory response was assessed through analysis of white blood cells count and globulin concentrations on the day of vaccination (day 0) and up to 7 additional visits or 270 days after vaccination depending on disease progression and survival (Figures 2A and 2B). One dog (AA06) had a leukocytosis (15.95 × 10^3^/mL) on the day of vaccination, with the remaining dogs having values within normal range (4.9–15 × 10^3^/mL). Over time, the median white blood cells count remained in normal range. Three dogs A004, A005, and A006 (37.5%) had an elevated globulin value on day 0 (3.7 g/dL), whereas other dogs had globulin value in normal range (2.7–3.5 g/dL). The median globin value remained in the normal range throughout the study.

### Hepatic function

Values for hepatic enzymes (aspartate aminotransferase [AST], alkaline phosphatase [ALP], and alanine aminotransferase [ALT]) were evaluated between day 0 and until end of the study (Figures 2C and 2E). One dog (AA05) had an elevated AST value on the day of vaccination with the remaining dogs having values within normal range. The median AST value on the day of vaccination was 22 U/L (range 19 U/L to 51 U/L). AA05’s AST value had increased in the 14 days following vaccination (Figure 2C), and the dog was removed from the study due to disease progression.

Four dogs A003, AA05, AA06, and A008 (50%) had a persistently elevated ALP (range from 155 to 398 U/L) values from day 0 to the end of the study, with the remaining four dogs having values within normal range (range from 8 to 139 U/L) (Figure 2D).

Median ALT value remained within normal range throughout the duration of the study (10–92 U/L). Similarly, three dogs (37.5%) A003, AA05, and AA08 had elevated ALT values on the day of vaccination (from 96 to 103 U/L), with the remaining five dogs having values within normal range (Figure 2E). The median ALT value was 68 U/L (range, 23 U/L to 103 U/L) on the day of vaccination and 91 U/L (range, 27 U/L to 145 U/L) 14 days later. Four dogs (4/8, 50%) had elevated ALT values 14 days after vaccination.

### Renal function

Renal function was assessed through analysis of creatinine and blood urea nitrogen (BUN). All eight dogs had normal creatinine and BUN values on day 0 (Figure 2F), with a median value of 1.0 mg/dL and 17 mg/dL, respectively, and the values remained generally unchanged at through duration of study, except for A006 that had elevated BUN on day 14 and 28.

### Cellular immune response against tumor antigens

**Figure 3:**
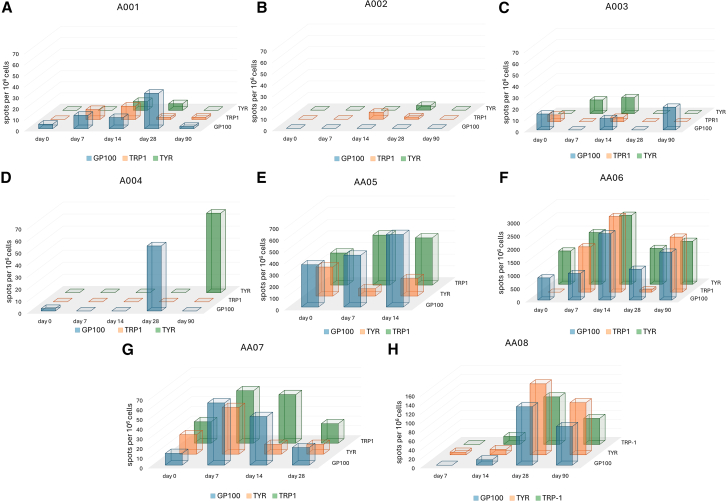
Kinetics of immune responses to vaccine delivered tumor antigens evaluated in IFN-γ ELISPOT assay The dogs’ PBMCs collected at different time points were used to analyze the number of IFN-γ producing cells in response to stimulation with the pools of peptides from the three melanoma antigens used in the vaccine. Responses were divided into three groups according to time points of IFN-γ production: low responders (A–C), early responders (D–G) and late responders (H). Graphs were created by counting the number of spots in samples stimulated with peptides for 48 h and then subtracted the number of spots in non-stimulated samples to account for background activation of cells.

Analysis of IFN-γ production by circulating T cells re-stimulated with the pool of peptides for each tumor-associated antigen (TAA), GP100, Tyr, and TRP-1, was performed by canine IFN-γ ELISPOT assay. All dogs but one (87.5%) had a positive response to at least one TAA at one time point (Figure 3). Only one dog (A002) did not present the response to any of the 3 TAA at any time point measured. The absence of response in dog A002 is likely associated with an overall weak immune system due to advanced age (13.1 year), advanced disease stage, or low immunogenicity of peptides presented by the dog’s specific HLAs. 5/8 (62.5%) of the dogs presented a response to at least one TAA on day of vaccination suggesting immune activation due to local therapy, similar to human clinical trials.^51^ In three dogs (AA06, AA07, and AA08, 37.5%), the strong response to all 3 TAAs was presented at several time points. IFN-γ production was not detectable at 2 months or later after vaccine administration.

### Humoral immune response against the vector capsid

The initial evaluation of titers against AAV capsid was performed as part of the inclusion criteria. All dogs enrolled in the trial had a neutralizing antibodies (NAb) titer <1:2, below the threshold for inclusion of 1:8. Additionally, NAb titers were monitored at each visit to evaluate the kinetics of the humoral response to the AAV capsid (Table 2). In all eight dogs, we were able to detect the generation of NAb in response to AAV administration but with different degrees. In two dogs (A002 and A003), the titers started to rise as early as 7 days post-AAV administration which might indicate that these dogs had prior exposure to AAV and this is a secondary response. In other dogs, NAb elevated between day 14 and 28 after vaccination. One dog (A001) showed NAb titers <1:8 at every time point up to day 150 of monitoring. In most dogs, the newly generated NAb declined over time. More importantly, in some dogs the NAb titers returned to background level of <1:2 creating the possibility to re-administrate the same AAV vaccine. In one dog (A008), a high NAb titer maintained until the end of the study (day 270).

**Table 2:** Summary table of NAb assay

NAb titer	Time −1	Injection	Day 7	Day 14	Day 28	Day 90	Day 150	Day 210	Day 270
A001	1:2	1:2	1:2	1:2	1:7	1:2	1:2	–	–
A002	1:2	1:2	1:50	1:185	1:168	1:2	–	–	–
A003	1:2	1:2	1:40	1:79	1:360	1:131	1:2	1:8	1:2
A004	1:2	1:2	1:2	1:83	1:211	1:207	1:192	1:61	1:32
AA05	1:2	1:2	1:2	1:2	–	–	–	–	–
AA06	1:2	1:2	1:2	1:28	1:405	1:418	–	–	–
AA07	1:2	1:2	–	1:2	1:12	1:33	–	–	–
AA08	1:2	1:2	1:2	1:2	–	1:296	–	1:210	1:147

The dogs’ serum was assayed to check for NAb against the AAV capsid. The NAb titer was calculated using a serial dilution of the serum and exposed it to AAV6-S663V virus containing luciferase protein. The luciferase signal decreases when exposed to antibodies in the serum. The sigmoidal curve representing the serial dilution showed when the luciferase signal decreased by 50%, which is the equivalent of the antibody titer for each sample. Two dogs did not show NAb against the vaccine vector. The rest of the animals had detectable NAb that reached a maximum, in most animals, peak between day 14 and 150 after vaccination. Missing data points are due to no attendance by the patient or removal from study.

### Rechecks and survival time

All eight dogs had their disease status assessed at each recheck visit along with thoracic radiographs, if indicated by the study protocol. Three dogs (A003, A004, and AA08; 37.5%) completed the study without any evidence of local or systemic progression, and two of them (A003 and A004) survived for more than 2 years after vaccination (Table 1). A total of five dogs (62.5%) were removed from the study due to either local or systemic progression. Three of these five dogs (37.5%) developed pulmonary metastasis. These five dogs developed disease progression as early as 14 days after vaccination, with a median of 113 days; the median survival after progression was 328.8 days. None of the dogs had a postmortem evaluation.

## Discussion

Our study examined, for the first time, the use of an optimized AAV-based cancer vaccine expressing three tumor antigens (GP100, Tyr, and TRP-1) in a spontaneous canine cancer model, particularly oral melanoma. These melanoma antigens were used due to their highly conserved protein sequences and function across multiple species, which allowed for translation of our studies from the B16F10 mouse model^16^^,^^17^ directly to dogs, and with possibility for a straightforward translation to humans without changing vaccine design.

Our study was designed as a single-arm open-label trial that relies on differences of before-after treatment parameters, comparisons with historical controls, and outcomes of other published trials. Ethical aspects were also taken into consideration.

To increase the possibility of favorable clinical outcomes, the dog`s owners were able to elect local therapies such as surgery and/or radiotherapy before the administration of the optimized AAV vaccine (Table 1). Median survival times (MST) have been reported to be up to two years with adequate local control versus only a few months without, acknowledging that this can vary depending on stage of disease at diagnosis and histopathological features.^52^ Similar treatment protocol applies to human patients which allows immune response to control possible metastatic spread and recurrence at primary site rather than overcome “cold” tumor microenvironment.^53^^,^^54^^,^^55^^,^^56^^,^^57^^,^^58^^,^^59^^,^^60^ Our data indicate, as observed with patients A003, A004, and AA08, that early diagnosis and treatment (surgery, radiotherapy, and vaccination) favor survival and outcome, as seen in other studies.^61^ Two dosages, 3 × 10^12^ or 9 × 10^12^ vg/dog were selected based on conversion of doses used in mouse studies^17^^,^^24^ and administered intramuscularly. These doses were much lower than generally required for dog’s and human gene therapy studies because for vaccine purposes it is enough to deliver genetic cargo content to a small population of antigen-presenting cells by local administration to elicit a sufficient immune response. In addition, the optimized AAV vaccine used in our studies is specifically designed to amplify antigen-specific immune responses.^15^^,^^16^^,^^17^ For example, in gene replacement therapy for golden retriever muscular dystrophy^41^ aiming to correct genetic defects, very high doses were required to achieve a therapeutic effect, ranging from 5 × 10^12^ to 5 × 10^13^ vg/kg in 3- to 4-month-old dogs. In another example, gene therapy for companion dogs with Hemophilia B required 6 × 10^12^–5 × 10^13^ vg/kg to achieve therapeutic effect.^36^ The advantage of reduced AAV dose is to avoid triggering adverse side effects associated with high vector load.^62^

It has been shown that AAV-based therapies can induce a significant inflammatory response that severely affects the function of the liver and kidneys, typically within the first 2 weeks after administration.^63^^,^^64^ We were able to detect this response to AAV by observing relative elevation of common inflammatory markers MyD88, TLR9, IFN-γ, TNF-α, and IL-6, previously reported as indicators of AAV infection,^65^ with quantitative reverse-transcription PCR (RT-qPCR) performed for PBMCs collected 2 h after vaccination on three tested dogs. This immediate post-vaccination elevation was not associated with noticeable adverse reactions since other parameters, such as, total WBC count, globulins, as well as the liver and retinal median values remaining within normal range during the course of the study. These data demonstrated that none of the vaccinated dogs were presented with major adverse events or side effects, not immediately after vaccine infusion nor on a long term, indicating that both doses were well tolerated (Table 1).

Additionally, we observed an anticipated increase in NAb titers against AAV capsid in almost all animals, with peaks between 1 and 4 weeks after vaccine administration regardless of doses (Table 2). Importantly, for some of the dogs (A003 and A004) AAV NAb titers declined to background levels at later points. These data suggest that in some cases, the same AAV serotype can be re-administered, particularly for the purpose of immune response boosting.

The vaccination-induced antigen-specific response was analyzed by IFN-γ ELISPOT assay with PBMCs isolated from blood at different time points. The analysis demonstrated a significant increase in levels of antigen-specific T cells against at least one antigen for each dog and in general all three antigens (GP100, Tyr, and TRP-1) demonstrated immunogenicity at least in one of the dogs (Figure 3). These data support the benefits of the triple valent vaccine and confirm that the use of sequence conservative tumor-antigens for cancer vaccines is a viable option for dogs, as it is for humans,^51^ allowing the use of these antigens as treatment option for patients with oral and other types of melanomas. Since peptides in the pools for all three antigens can be presented by MHC-I and MHC-II class molecules, we cannot specify T cells subpopulation primarily responsible for IFN-γ production in ELISPOT. However, in our previous studies in mice where individual immunodominant peptides for MHC class I and MHC class II were available, we showed that both CD8^+^ and CD4^+^ T cells produced IFN-γ. Among immunotherapies, the Oncept xenogeneic DNA vaccine (targeting human tyrosinase) and CSPG4-targeted DNA vaccines have received significant attention.^49^^,^^66^ While some studies report improved survival with adjuvant vaccination, others show mixed or inconclusive results, especially regarding Oncept.^49^^,^^67^ Prognosis is influenced by tumor stage, size, completeness of excision, bone invasion, and presence of metastasis.^68^ Oncepts’ early prospective trials suggested improved MST of more than 437 days vs. historical controls at 324 days when used as adjuvant therapy after local control.^69^^,^^70^ At the same time, several retrospective studies failed to show a statistically significant improvement in progression-free or overall survival compared to non-vaccinated controls.^67^^,^^71^^,^^72^^,^^73^^,^^74^^,^^75^ Regarding CSPG4-targeted DNA vaccines, studies show a significantly prolonged MSTs (684–1,333 days) compared to surgery alone (200–470 days), with lower metastatic rates,^61^^,^^76^^,^^77^^,^^78^^,^^79^ while dendritic cell-based vaccines preliminary studies indicate safety; some dogs achieve stable disease or prolonged progression-free intervals when combined with radiotherapy.^61^

Thus, in comparison with existing vaccine-based approaches, the outcome of current pilot studies is highly encouraging, demonstrating that five out of eight dogs survived more than a year with three of eight dogs (37.5%) without recurrence of primary tumor or lung metastasis, therefore disease-free at the end of the study with the overall MST for all eight dogs after disease progression was 556.25 days, two dogs have lived disease free for over 804 days; one dog was still alive while this manuscript was prepared, having survived more than 1,044 days after vaccination.

Limitations of this study include inconsistencies in the dogs’ initial WHO stage (enrolled patients were at different stages), initial thoracic imaging, the presence of gross disease at the time of vaccination, and the lack of postmortem evaluation in any of the deceased dogs. WHO stage has been documented in multiple studies^52^^,^^80^^,^^81^^,^^82^ to be a prognostic factor in dogs with oral melanoma. Therefore, the initial WHO stage seems to have influenced the survival data. Additionally, there were some dogs that had gross disease present at the time of vaccination. At this time, it is not known whether our vaccine might work better in the microscopic disease setting than for gross disease, and the presence of gross disease could have influenced the survival data and led to euthanasia sooner for these animals than for pets who did not have gross disease initially. The dogs were also variable ages at the time of vaccination (Table 1), and the oldest dog was over 14 years old (A001).

Even with the limited number of canine patients enrolled, this study provides valuable information about safety. The preliminary conclusions resulting from our study indicate that off-the-shelf AAV vaccine based on common melanoma antigens is safe in dogs, which opens the possibility of expanding the repertoire of diseases that benefit from AAV vector-based therapy.

## Materials and methods

### Case selection

Canine patients were referred to the University of Minnesota’s (UMN’s) Veterinary Oncology department with a diagnosis of malignant oral melanoma. The medical record was reviewed by a veterinary oncology resident and an American College of Veterinary Internal Medicine board-certified veterinary oncologist. The patients were assessed, and further standard-of-care treatment recommendations were discussed. The possibility of enrolling in the AAV clinical trial was presented as one of the available options, when appropriate.

Dogs were eligible to enroll if they had oral melanoma confirmed via biopsy, were at least 1 year of age, and weighed at least 5 kilograms (approximately 11 pounds). Dogs were only included if their life expectancy was at least 6 weeks. Patients were also eligible if they had evidence of local recurrence of their oral melanoma or if they had confirmed locoregional lymph node involvement. They were excluded if there was evidence of distant metastasis beyond the locoregional lymph nodes (e.g., to the lungs), or any severe comorbidities based upon review of their previous medical history and pre-enrollment lab work (complete blood count, chemistry panel, urinalysis). Dogs were excluded if they had received any corticosteroids or immunosuppressive drugs within 7 days of enrollment or if they had previously received the Oncept melanoma vaccine. All dogs were tested for the presence of NAb against AAV6 and excluded if they had NAb titers greater than 1:8.

### Enrollment procedures

Dogs were screened to ensure they met all eligibility criteria. Screening included blood collection (for complete blood count and chemistry panel performed at the UMN Clinical Pathology Lab and evaluation of NAb titers against AAV performed by the Aslanidi lab) and urine collection (for urinalysis performed at the UMN Clinical Pathology Lab). If deemed eligible, dogs were either scheduled for advanced imaging diagnostics including a (CT) scan of the skull, neck, and thorax performed under general anesthesia or had thoracic radiographs performed (when owners declined CT). Fine needle aspirates of locoregional lymph nodes (e.g., mandibular, prescapular) were submitted for cytology and were reviewed by a board-certified veterinary clinical pathologist even if they were normal in size. After staging, dogs underwent appropriate local therapy (surgery or radiation therapy, depending on owner preference), if not already performed prior to vaccination.

The clinical trial was conducted with IACUC oversight and approval (IACUC protocol # 2111-39555A, 2409-42382A).

### Vaccination procedures and clinical monitoring

Approximately 2 weeks after surgery/radiation therapy or 7 days after NAb titer collection (if local therapy had been performed prior to referral or owner elected not to perform any), blood was collected for complete blood count and a chemistry panel, urine was collected for urinalysis, and a physical examination was performed by a UMN Oncology clinician. The AAV vaccine was administered intramuscularly into the quadriceps muscles in two dosages, 3 × 10^12^ vg/dog or 9 × 10^12^ vg/dog, and the dog patients were observed 2 h after injection for any evidence of a vaccine reaction, including but not limited to facial swelling, urticaria, vomiting, or breathing difficulties.

Recheck examinations were performed 7 and 14 days after vaccination, which included a physical examination; blood collection; urine collection; and assessment of energy level, appetite, and behavior. At 28 days after vaccination, thoracic radiographs were performed in addition to the aforementioned diagnostics. Recheck examinations at 3, 5, 7, and 9 months were performed with the same procedures as the day 28 examination. Unscheduled visits were performed if there were concerns expressed by the owner (related to side effects or quality of life) or when repeat blood collection was needed.

### Data collection

Data were collected on breed, sex, age, body weight, location of melanoma, melanoma stage according to the World Health Organization^83^ prior to local therapy, disease status at each recheck evaluation, local or systemic progression, and cause of death. Laboratory values were recorded on day 0 (day of vaccination) and day 14 to further assess safety of our vaccine; most of the side effects observed in the clinical application of AAV treatments are associated with hepatic toxicity or renal failure due to inflammatory processes.^63^^,^^84^^,^^85^ White blood cells count, globulins, ALT, (ALP, AST, creatinine, and BUN were measured.

### AAV vaccine design and production

The truncated melanoma antigens GP100, Tyr, and TRP-1, coupled with MHC class I trafficking signals as previously described,^16^^,^^17^^,^^24^ were cloned in a self-complementary expression cassette^86^ driven by the CMV promoter and packaged in AAV6 containing a single mutation in the VP3 capsid protein at amino acid position 663 to substitute serine (S) with valine (V).^87^ Each vector was packaged in HEK293 cells with a triple transfection protocol. Packaging plasmids were isolated with an endotoxin-free purification kit (Takara) to ensure an endotoxin level below 0.02 EU/mL. Vectors were purified by iodixanol gradient followed by HiTrap HP ion-exchange column purification as previously described.^16^ Chromatography purification was conducted on an ÄKTA Pure chromatography system operated by UNICORN software version 7.5 (Cytiva, Piscataway, NJ, USA). The material eluted from HiTrap HP was collected by a fraction collector (Cytiva). The fractions with the most abundant vg presence were concentrated by using spin concentrators with 150 kDa molecular weight cut-off (#AP2015010, Orbital Biosciences, Topsfield, MA, USA). Final AAV products were stored in sterile phosphate-buffered saline (PBS), pH 7.0. Viral genome titers were determined by qPCR with a TBGreen Advantage (#S4748, Takara Bio, San Jose, CA, USA) and primer pair specific to the CMV promoter within the AAV expression cassette. The vectors mixed in 1:1:1 (1 × 10^12^ vg/antigen/dog or 3 × 10^12^ vg/antigen/dog respectively) ratio in a total volume of 1–2 mL in two doses: 3 × 10^12^ vg/dog and 9 × 10^12^ vg/dog.

### Blood PBMCs and plasma extraction

PBMCs composed of T cells, B cells, monocytes, neutrophils, etc were collected from whole blood on all time points of veterinary visits for IFN-γ ELISPOT assay. PBMCs were collected twice on day of vaccination (prior to vaccination and 2 h after vaccine administration in the high-dose cohort) for analysis of the inflammatory markers IFN-γ, TNF-α, IL-6, MYD88, and TLR9. Ten milliliters of blood were collected in EDTA tubes. The blood was transferred to a 15-mL conical tube and centrifuged at 2,000 rpm for 10 min. The plasma from the top layer was separated and frozen at −20°C. The remaining blood was mixed with PBS at a 1:1 ratio, transferred to a Lymphoprep tube (PROGEN), and centrifuged at 800 *g* for 20 min. The PBMCs above the mesh layer were transferred to a 15-mL conical tube and centrifuged at 800 g for 10 min. Pelleted PBMCs were resuspended in 10 mL of 1× RBC Lysis buffer (BioLegend), incubated for 5 min, and centrifuged at 800 *g* for 10 min. This step was repeated up to three times until PBMCs were visibly clear of remaining red blood cells. PBMCs were stored in freezing medium (90% fetal bovine serum [FBS; Gibco]) + 10% dimethyl sulfoxide ([DMSO], Millipore-Sigma) in a −80°C freezer.

### IFN-γ ELISPOT

Canine ELISPOT IFN-γ (Mabtech) was used to assay the dogs’ PBMCs according to manufacturing instructions. Briefly, PBMCs were thawed, counted in CellDrop (DeNovix), and a total of 0.2–0.5 million cells/well in RPMI medium with 10% FBS were plated on 96-well Multiscreen IP sterile plate (Millipore-Sigma) coated with 15 μg/mL anti-canine IFN-γ monoclonal antibody (mAb; MT13). Cells were treated with 100 μg/mL of custom pools of overlapping peptides (each peptide is 15 aa with 11 aa overlap) covering the full sequences of canine GP100, Tyr and TRP-1 (GeneScript), 20 ng/mL of recombinant canine IL-2, (400-11, Shenandoah Biotechnology) and 20 ng/mL IL-21, (ICNIL21R, Innovative Research). The plate was incubated for 48 h at 37°C. The plate was treated with a biotin-conjugated anti-canine IFN-γ mAb (MT166) for 2 h at RT followed by streptavidin ALP antibody for additional 1 h at RT. BCIP/NBT-plus substrate solution was used to visualize spots. The plate was stored in the dark overnight to let it dry, and spots were counted the next day in an ELISPOT reader (R&D Systems).^16^^,^^17^^,^^24^ Results are presented as the number of spots per 10^6^ cells.

### Anti-AAV neutralizing antibody assay

The protocol has been previously described.^87^^,^^88^ Briefly, HEK293 cells were plated in a 96-well plate and grown overnight at 37°C. HEK293 cells were pre-treated with 10 μM of Compound C (Millipore-Sigma) for 1 h at 37°C. The serum was diluted 1:2, 1:8, 1:32, 1:128, and 1:512 in heat-inactivated FBS and mixed with AAV6-CB-fLuc, added to the HEK293 cells, and incubated at 37°C. The luciferase activity was estimated 48 h later by using the chemiluminescence produced by the degradation of the Bright-Glo (Promega) substrate. The NAb titer was estimated as a 50% inhibition of chemiluminescence, as previously described.^29^^,^^89^

### qPCR of inflammatory cytokines

A panel of inflammatory cytokine genes was assayed to evaluate the acute response to AAV vaccine administration 2 h after vaccination. PBMCs were extracted at the time of AAV vaccine injection and again 2 h later. A Quick-RNA MiniPrep Plus kit (Zymo) was used for RNA extraction following the manufacturer’s instructions. Protoscript II first-strand cDNA synthesis kit (New England Biolabs) was used to synthesize cDNA from 500 ng of RNA template. qPCR was performed on the cDNA template with a TBGreen Advantage (#S4748, Takara Bio, San Jose, CA, USA) and the following primers: cIFN-γ F-gattctgactccttttccgct and R-tagcagcaccagtaagaggg, cTNF-α F-ctgaaagcatgatccgggac and R-agggctgattagttggaggcc, cIL-6 F-ctactgctttccctaccccg and R-gcctctttgctgtcttcaca, cMYD88 F-gaggagtctgagaagccctt and R-cacacacaacttcagccgat, and cTLR9 F- cggtggaagcaggtggag and R-agagggtctggctggcag.

## Data and code availability

Data and materials described in this manuscript will be available upon request to the corresponding author.

## Acknowledgments

This project was supported by NIH/NCI R01CA285620 grant (GA and AB), a Paint the Town Pink pilot grant (GA), startup funds from the Hormel Foundation (GA), and a Fifth District Eagles Cancer Telethon Postdoctoral Fellowship Award (HK and FG). We would like to thank veterinarians and staff at Clinical Investigation Center, University of Minnesota Veterinary School, for support of our study. We particularly would like to thank the dog`s families for participation in our trial.

## Author contributions

K.K., A.B., and G.A. developed the concept of the project and designed experiments. F.G., E.M., C.Y., C.C., and H.K. performed experiments. F.G., C.Y., E.M. analyzed data. F.G., E.M., C.Y., K.K., A.B., and G.A. wrote the manuscript. C.C. and H.K. edited the manuscript. G.A. and A.B. obtained funding and supervised the project. All authors agreed with content presented in the manuscript.

## Declaration of interests

G.A. and K.K. have several issued or provisional patents related to AAV vectors that have been licensed to various gene therapy companies.
